# Mapping Eco-Affective Health: A Spatial Framework for Climate-Related Emotional Responses and Mental Health in Urban Systems

**DOI:** 10.3390/ijerph23080991

**Published:** 2026-07-29

**Authors:** Lucas Murrins Marques

**Affiliations:** 1 Mental Health Department, Santa Casa de São Paulo School of Medical Sciences, São Paulo 01238-010, SP, Brazil; lucas.murrins@fcmsantacasasp.edu.br; 2 Eco-Wellbeing & Affective Health Laboratory (EWAH Lab), São Paulo 01238-010, SP, Brazil

**Keywords:** eco-affective health, climate-related emotions, eco-anxiety, solastalgia, planetary health, environmental exposure, public mental health, urban systems, spatial analysis, resilience

## Abstract

**Highlights:**

**Public health relevance—How does this work relate to a public health issue?**

- Climate change and urban environmental degradation are now recognized as central determinants of population mental health, acting through spatially structured exposure pathways.
- Climate-related emotional responses, including eco-anxiety, solastalgia, and ecological grief, emerge from the interaction between environmental exposures, landscape configuration, and individual vulnerability within urban systems.

**Public health significance—Why is this work of significance to public health?**

- The Eco-Affective Health Mapping (EAHM) framework is proposed as a conceptual tool for the spatial identification of populations that may be at elevated risk of climate-related mental health burden, intended to support, pending empirical validation, proactive public health surveillance and intervention.
- By integrating environmental exposures, landscape structure, and affective indicators within a unified spatial framework, EAHM advances the field of eco-affective health as a theoretically grounded and, in principle, measurable and actionable public health construct, building on the companion Eco-Affective Health theoretical framework and assessment protocol referenced throughout this article.

**Public health implications—What are the key implications or messages for practitioners, policymakers and/or researchers in public health?**

- EAHM provides a practical tool for urban planners and public health policymakers to identify eco-affective vulnerability hotspots and design spatially targeted mental health resilience interventions.
- The framework is designed to be applicable across diverse socioeconomic contexts, including data-limited settings in the Global South, supporting equitable and prevention-oriented public mental health strategies.

**Abstract:**

Urban environments concentrate spatially distributed stressors, including heat, noise, pollution, and biodiversity loss, that are increasingly recognized as determinants of population mental health. However, current approaches rarely integrate environmental structure, lived exposure, and climate-related emotional responses within a unified spatial framework applicable to public health. This article introduces Eco-Affective Health Mapping (EAHM) as a conceptual, spatially explicit framework, grounded in the recently formalized Eco-Affective Health theoretical model, for understanding how environmental conditions may shape climate-related emotional responses, including eco-anxiety, solastalgia, and ecological grief, across urban socio-ecological systems. Drawing on evidence from spatial epidemiology, landscape ecology, environmental mental health, and digital phenotyping, I argue that affective responses to environmental stressors are not randomly distributed but are hypothesized to exhibit spatial clustering in relation to environmental exposures, landscape configuration, and mobility-based interactions. I propose the Eco-Affective Health Mapping (EAHM) framework, which integrates four spatial layers: environmental exposures, landscape configuration, person–place interaction, and affective indicators, together with a companion composite metric, the Eco-Affective Load Index (EALI), for which I provide a formal multi-domain specification and a purely illustrative, non-empirical worked example. EAHM is presented here as a theoretical and methodological proposal rather than as a validated instrument: no primary environmental, mobility, or affective data were collected or analyzed for this article, and the framework’s constituent relationships require prospective empirical testing, for which I outline a companion measurement strategy grounded in the Eco-Affective Health Assessment Protocol (EAHAP). By conceptualizing climate-related emotional responses as candidate measurable public health signals, EAHM is intended to support a future shift toward prevention-oriented, population-level mental health strategies aligned with planetary health and sustainable development agendas.

## 1. Introduction

Climate change and environmental degradation are reshaping the mental health landscape of urban populations worldwide. Beyond their ecological and economic consequences, environmental stressors, including heat extremes, air pollution, noise, biodiversity loss, and flooding, are now recognized as significant determinants of psychological well-being, affective regulation, and population resilience [1,2]. A growing body of evidence documents the emergence of climate-related emotional responses, such as eco-anxiety, solastalgia, ecological grief, and soliphilia, as distinct affective states that reflect how individuals and communities perceive, process, and respond to environmental change [3,4]. These responses are not uniformly distributed across populations; rather, they exhibit spatial patterning shaped by the structure of urban environments, socio-spatial inequalities, and differential environmental exposures [5,6].

Urban systems are increasingly understood as complex socio-ecological environments in which environmental conditions and human responses co-evolve across space and time. From a public mental health perspective, this means that the spatial organization of cities, including the distribution of green and blue spaces, heat islands, noise corridors, and polluted zones, directly influences where and among whom climate-related mental health burdens accumulate [7,8]. Environmental noise, for example, has been shown to display spatially patterned associations with depression and anxiety, particularly when interacting with socio-economic deprivation gradients [9]. Similarly, urban heat island effects are associated with disruptions in psychological regulation, with spatial clustering of impacts observed in areas characterized by limited vegetation and shading [10].

At the same time, advances in digital and community-scale sensing are reshaping how climate-related emotional responses can be captured and analyzed at the population level. Ecological momentary assessment, smartphone-based digital phenotyping, and geospatial methods provide new opportunities to measure affective states in real time, linked to specific environments and mobility sequences [8,11,12]. These methodological innovations enable a shift from static, outcome-based models of environmental mental health toward dynamic frameworks that capture the continuous interaction between individuals, their environments, and their affective responses.

Despite this progress, two established research traditions have developed largely in parallel, and it is the gap between them that motivates the present framework. Environmental health mapping approaches, including GIS-based exposure surveillance, health impact assessment, and environmental-justice screening tools, systematically characterize the spatial distribution of biophysical stressors such as heat, noise, and pollution, but rarely incorporate affective or emotional indicators as part of the mapped output [7,9,10]. Conversely, the eco-affective and climate-emotion literature has produced psychometrically developed constructs such as solastalgia, eco-anxiety, and ecological grief [13,14,15], but has done so largely independently of spatial exposure science, typically using single-site, non-georeferenced self-report data collected without reference to landscape structure or person-place interaction [16]. Neither tradition alone provides an architecture capable of jointly representing where environmental stressors concentrate, how landscape structure moderates their psychological impact, how people are actually exposed through everyday movement, and how affective responses are consequently distributed across urban space. It is this specific integration gap, rather than a general absence of environmental mental health research, that the framework proposed below is intended to address.

Building on the emerging field of eco-affective health, I propose a spatially explicit framework for understanding how environmental stressors and restorative system components interact to generate patterned climate-related emotional responses across urban populations. In this framework, affective states are conceptualized not solely as individual psychological outcomes, but as emergent and measurable public health signals that reflect the balance between environmental stress and adaptive capacity at the population level.

This perspective integrates recent evidence on climate-related emotional responses, including research demonstrating that solastalgia exhibits geographic clustering in regions undergoing environmental change [3], with spatial evidence linking landscape configuration and environmental exposures to mental health outcomes [6,7]. In doing so, it contributes to the broader agenda of eco-affective health by providing a framework through which climate-related emotional responses can be incorporated into public health monitoring, urban planning, and resilience-oriented policy.

In this article, I synthesize the spatial logic of climate-related emotional responses within urban systems and introduce a mappable, four-layer Eco-Affective Health Mapping (EAHM) framework. The EAHM approach integrates environmental metrics, landscape configuration, mobility-based exposure, and affective indicators within a unified spatial analytical system. I discuss its theoretical grounding and its relationship to existing environmental-health mapping and eco-affective measurement traditions, a formal specification of its composite index, a companion measurement strategy, its applications for global public health, its implications for prevention-oriented mental health strategies, and its alignment with planetary health and sustainable development agendas.

## 2. From Planetary and Environmental Health to Eco-Affective Health

The transition from planetary health to environmental mental health has expanded understanding of how environmental conditions influence human psychological functioning and population well-being. Planetary health frameworks emphasize the interdependence between environmental stability and human health [17], while a growing body of evidence demonstrates that exposures such as heat, air pollution, noise, and biodiversity loss are associated with changes in affective regulation and adaptive capacity in urban populations [1,7].

Within this context, climate-related emotional responses have emerged as a distinct and clinically relevant domain of inquiry. Solastalgia, defined as distress associated with environmental degradation in one’s home environment, has been documented across diverse cultural and geographical settings, with evidence of geographic clustering in regions undergoing rapid ecological change [3]. Eco-anxiety, characterized by persistent worry about environmental futures, has been shown to affect individuals across age groups and socioeconomic strata, with particular intensity among those with high environmental awareness or direct exposure to climate-related events [4]. Ecological grief and soliphilia, a sense of love and responsibility toward one’s place and ecosystem, further expand the affective spectrum through which individuals relate to environmental change [2].

These climate-related emotional responses are not isolated psychological phenomena. Evidence indicates that they are shaped by the same environmental exposures and landscape configurations that influence broader mental health outcomes. Reduced access to green and blue spaces has been consistently associated with increased stress, reduced restoration capacity, and diminished affective regulation, whereas ecologically rich and connected landscapes support improved psychological functioning and resilience [7,8]. Environmental noise and urban heat island effects generate spatial clusters of disrupted affective regulation, particularly in areas characterized by elevated exposure intensity and limited environmental buffering [9,10].

Advances in digital phenotyping and ecological momentary assessment provide new tools for capturing these dynamics at the population level, allowing climate-related emotional responses to be measured as time-varying outputs of environmental exposure and mobility within urban environments [8,11,12]. These methodological developments support the integration of eco-affective states into public health surveillance systems and mental health monitoring frameworks.

Within this context, I define Eco-Affective Health as an extension of environmental mental health that conceptualizes climate-related emotional responses as spatially structured public health indicators. In this framework, affective dynamics, including eco-anxiety, solastalgia, ecological grief, and restorative responses, serve as measurable signals of how environmental conditions influence mental health and adaptive capacity across urban populations. This perspective positions eco-affective health as a necessary and operational construct for public health research, surveillance, and intervention.

### 2.1. Existing Environmental Health Mapping Approaches and Their Limits

Because the novelty claimed for EAHM depends on how it relates to existing spatial approaches, it is useful to situate it explicitly against them, rather than presenting it as a wholly unprecedented mapping exercise.

Environmental health mapping has an established tradition. The World Health Organization’s guidance on urban green space integrates exposure mapping with population health outcomes [1], and environmental-justice screening tools, together with health impact assessment methodologies, have long combined GIS-based exposure layers (air quality, noise, heat, flood risk) with demographic vulnerability indices to prioritize intervention areas [7,9,10]. Within environmental epidemiology, spatial analyses of noise and depression [5] and of urban landscape ecological risk and mental health [6] demonstrate that biophysical exposure metrics can be meaningfully linked to population mental health outcomes at fine spatial resolution. These traditions are methodologically mature but share a common limitation for the purposes of this article: they characterize exposure and, at most, diagnosed or self-reported mental health outcomes, without incorporating the intermediate affective constructs, eco-anxiety, solastalgia, ecological grief, soliphilia, that the eco-affective health literature has identified as mechanistically distinct from generalized psychological distress [13]. Conversely, the psychometric literature on eco-affective constructs has advanced considerably, producing validated instruments such as the Hogg Eco-Anxiety Scale [14] and the Climate Anxiety Scale [15], and a substantial scoping review of solastalgia measurement [18], together with a more recent systematic review of solastalgia’s correlates and consequences [19]. This literature builds on foundational work on solastalgia and ecological grief as distinct affective responses to environmental loss [20,21], and on the broader taxonomy of climate emotions [22,23], but has developed largely without spatial-analytical integration: administration is typically survey-based and non-georeferenced, and landscape structure and person-place interaction are rarely treated as first-class analytical variables. EAHM’s contribution is therefore not the introduction of new exposure metrics or new affective constructs, but the proposal of a shared spatial architecture within which established exposure-mapping methods and established (or companion) eco-affective instruments [16] can be integrated and jointly interpreted, a combination that, to my knowledge, does not currently exist as a formalized framework.

### 2.2. Distinguishing Eco-Affective Health from Adjacent Constructs

A related concern is conceptual rather than methodological: what, precisely, does Eco-Affective Health add beyond existing constructs such as environmental mental health, ecopsychology, and socio-ecological resilience? I address this directly, drawing on the companion theoretical treatment of the framework [13]. Environmental mental health, as commonly used in the epidemiological literature, treats affect as a downstream outcome of environmental exposure, to be predicted from exposure metrics and demographic covariates. Eco-Affective Health, by contrast, positions affect as a primary regulatory mechanism, that is, as a variable that determines what environmental information is noticed, how it is interpreted, and whether it motivates behavioral or protective response, rather than as a terminal outcome measured only after the fact [13,24,25]. This is not a semantic distinction: it implies that eco-affective indicators should be measured and modeled as leading, not merely concurrent or lagging, signals. Ecopsychology, in turn, is a broader interdisciplinary and often therapeutic tradition concerned with the psychological dimensions of the human-nature relationship; it has generated rich theoretical and clinical insight but has not, on the whole, produced a spatially explicit, population-level surveillance architecture of the kind proposed here. Socio-ecological resilience frameworks focus on system-level adaptive capacity, typically operationalized through institutional, economic, and ecological indicators, and generally do not include individual- or population-level affective states as primary measured variables, treating human well-being, when included at all, as an aggregate outcome rather than a signal layer with its own internal structure (eco-affective, cognitive, and functional domains, as specified in the companion assessment protocol [16]). EAHM inherits the affect-as-signal positioning of Eco-Affective Health and applies it specifically to the spatial domain, which is the respect in which it differs from all three adjacent traditions rather than from any single one of them.

## 3. The Spatial Logic of Climate-Related Emotional Responses

Climate-related emotional responses within urban environments follow a spatial logic shaped by environmental exposures, landscape organization, and mobility patterns. From a public mental health perspective, this spatial structure is significant because it indicates that the risk of eco-anxiety, solastalgia, and related affective states is not uniformly distributed across urban populations, but is systematically associated with the environmental conditions of specific places and neighborhoods [5,6].

Spatial epidemiology has demonstrated that vegetation coverage, air quality, biodiversity, noise, and heat exhibit territorial gradients that correspond to gradients in population vulnerability and mental health outcomes [1,7]. Higher access to green and blue spaces is consistently associated with improved affective regulation, reduced stress, and enhanced restoration capacity, suggesting that such environmental features function as protective factors for eco-affective health at the population level [7,8]. In contrast, monotonous, fragmented, or highly polluted landscapes are associated with reduced restoration potential and increased psychological strain, conditions that may amplify climate-related emotional responses such as eco-anxiety and ecological grief [2,4].

Environmental noise exhibits dose-response relationships with disruptions in affective regulation and mental health outcomes, mediated by local residential density and socioeconomic conditions [9]. Similarly, urban heat islands generate spatial clusters of psychological vulnerability, particularly in areas with limited vegetation, shading, and access to cooling resources [10]. These patterns indicate that climate-related emotional responses are sensitive to the spatial distribution of environmental stressors across the urban fabric.

Person-place interaction further modulates these dynamics. Ecological momentary assessment studies demonstrate that affective states fluctuate as individuals move through environments with varying sensory loads and ecological affordances [11,12]. From a population health perspective, this suggests that climate-related emotional responses are not static attributes of individuals but dynamic outputs of continuous interaction between people and their surrounding environments, shaped by mobility, daily routines, and cumulative exposure patterns.

At the neighborhood scale, landscape configuration, including connectivity, fragmentation, and proximity to ecological features, predicts differences in affective restoration and psychological resilience [6]. Social and infrastructural characteristics, such as perceived safety, accessibility of public spaces, and social cohesion, further mediate how environmental exposures are translated into eco-affective burden at the community level [8].

**Figure 1:**
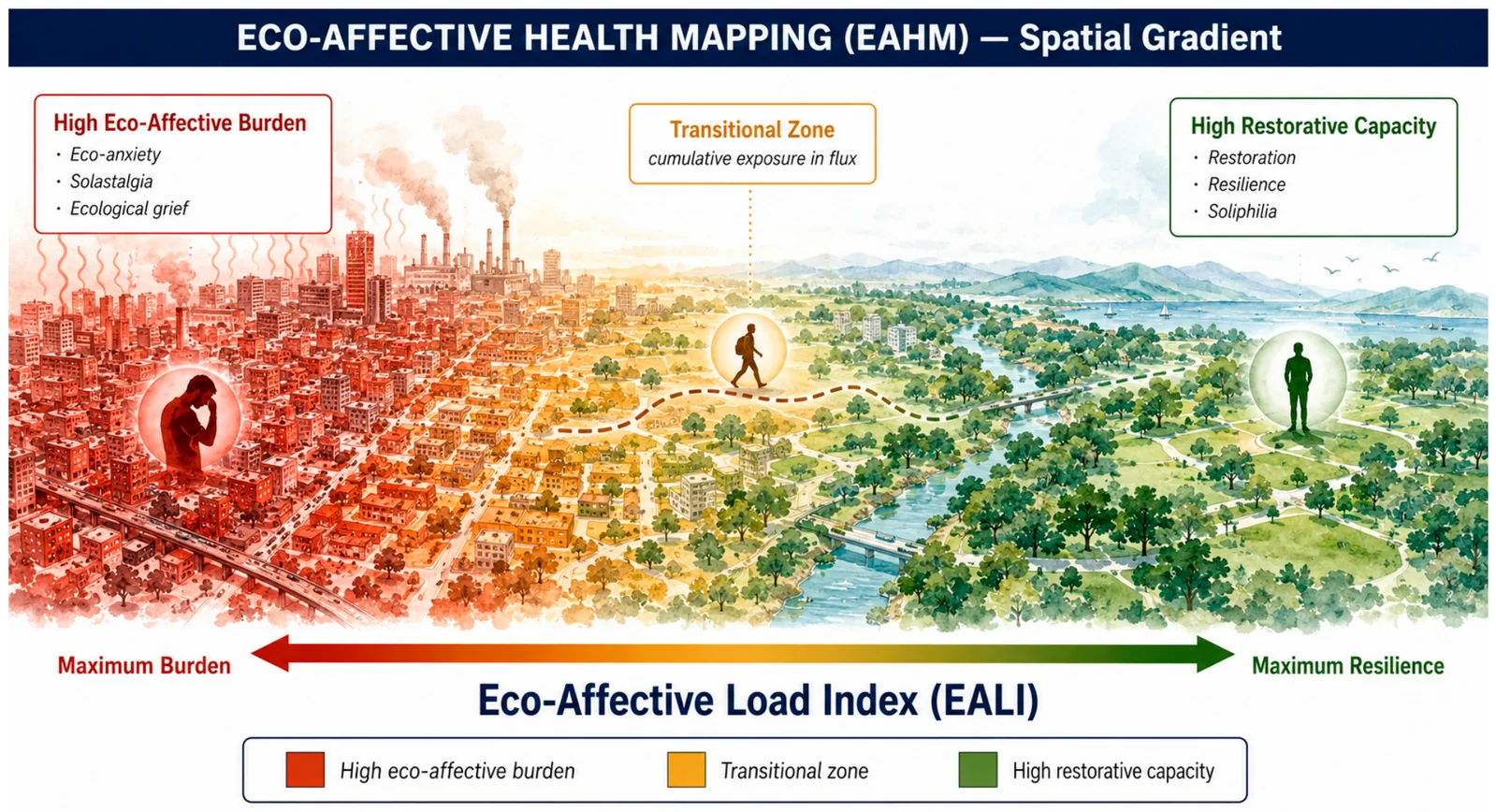
Spatial gradient of eco-affective health across an urban environment. The figure contrasts zones of high eco-affective burden (left), characterized by dense environmental stressors, landscape degradation, and heightened climate-related emotional responses, including eco-anxiety, solastalgia, and ecological grief, with zones of high restorative capacity (right), characterized by ecological connectivity, accessible green and blue spaces, and conditions supportive of restoration, resilience, and soliphilia. The central transitional zone reflects cumulative exposure in flux, illustrated by a person in movement navigating heterogeneous environmental conditions. The horizontal arrow represents the Eco-Affective Load Index (EALI) continuum, ranging from maximum burden to maximum resilience. Figure created by the author with the assistance of a generative AI image-generation tool (see Acknowledgments); conceptual structure and content specification are the author’s own.

In sum, climate-related emotional responses emerge from spatially patterned interactions between environmental exposures, landscape structure, and mobility dynamics. Recognizing this spatial logic is essential for public health, as it enables the identification of populations and places at elevated risk of eco-affective burden, and provides a basis for targeted, prevention-oriented interventions. Figure 1 illustrates this spatial gradient, contrasting zones of high eco-affective burden with zones of high restorative capacity across an urban environment.

## 4. Proposed Framework: Eco-Affective Health Mapping

I propose Eco-Affective Health Mapping (EAHM) as an integrative spatial analytical framework designed to capture how environmental conditions shape climate-related emotional responses and eco-affective health outcomes within urban populations. The framework is grounded in the premise that affective responses to environmental stressors are not randomly distributed, but exhibit spatial patterning that reflects the balance between environmental burden and restorative capacity at the population level [11,12].

The four-layer architecture described below is not an arbitrary partition of convenient variables; it follows the proximal-to-distal signal pathway formalized in the Eco-Affective Health framework [13]: environmental exposure conditions are filtered and moderated by landscape structure, are encountered by individuals through place-based interaction and mobility, and are ultimately registered as measurable affective and behavioral responses. Each EAHM layer corresponds to a distinct position in this pathway, consistent with the domain architecture already formalized for individual-level assessment in the companion Eco-Affective Health Assessment Protocol [16]. EAHM operationalizes this perspective by conceptualizing climate-related emotional responses as dynamic outputs of interactions between environmental exposures, landscape organization, and person-place dynamics. Within this framework, eco-affective states, including eco-anxiety, solastalgia, and ecological grief, are treated not solely as individual psychological outcomes, but as population-level signals that reflect how urban environments support or undermine mental health and adaptive capacity.

The framework is structured around four interacting layers. The first layer captures environmental exposures, including vegetation coverage, biodiversity, proximity to blue spaces, heat load, air pollution, and noise intensity. These variables represent key determinants of climate-related emotional responses and have been consistently associated with variations in eco-affective health across diverse urban contexts [7,8,9,10]. Data sources include remote sensing products, satellite-derived indicators, and municipal environmental monitoring systems.

The second layer addresses landscape configuration, focusing on the spatial arrangement and structural properties of environmental features. Metrics such as connectivity, fragmentation, patch size distribution, and edge density capture how ecological components are organized within the urban fabric. Evidence indicates that cohesive and connected landscapes support affective restoration and psychological resilience, whereas fragmented environments amplify eco-affective burden and reduce adaptive capacity [6,7]. In addition to these ecological-connectivity metrics, this layer incorporates built-environment variables that directly shape how stressors are experienced, including development density, building height and street-canyon geometry, and pedestrian-scale infrastructure such as sidewalk width and shading. These urban-form variables are included because landscape configuration is theorized to operate through two distinct, non-exclusive mechanisms: an autonomous restorative pathway, whereby exposure to green and blue features generates affective benefit largely independent of co-located stressor intensity (for example, visual contact with vegetation, which is associated with measurable reductions in stress and improvements in psychological restoration) [26,27,28,29], and a moderating pathway, whereby landscape structure attenuates or amplifies the psychological impact of co-located stressors (for example, tree canopy reducing perceived heat burden). EAHM does not assume these two pathways are equivalent or interchangeable; empirical applications of the framework should test them as distinct, separately measurable mechanisms rather than collapsing them into a single undifferentiated landscape score.

The third layer represents person-place interaction, capturing how individuals move through and engage with heterogeneous urban environments. Digital mobility traces and ecological momentary assessment data demonstrate that climate-related emotional responses vary as a function of exposure sequences, route characteristics, and time spent in specific environmental contexts [11,12]. This layer reflects the dynamic coupling between individual behavior and environmental structure that underlies eco-affective health at the population level. Because engagement with urban space is substantially shaped by the transportation and mobility infrastructure available to residents, this layer also incorporates indicators of transit accessibility, pedestrian-zone availability, cycling infrastructure, and route-level safety, alongside raw mobility-trace data. These structural features determine not only the intensity of environmental stress a person encounters, but the degree of agency they have to avoid or seek out particular environmental conditions in daily life, a distinction that mobility traces alone cannot capture.

The fourth layer incorporates affective and behavioral indicators, including self-reported eco-anxiety and solastalgia, perceived environmental threat (drawing on the established distinction between the affective and cognitive components of environmental risk perception [30,31]), restoration experience, physiological regulation markers derived from wearable devices, sleep variability, and passive behavioral signals from smartphones. These indicators provide measurable population-level outputs of how individuals experience and respond to environmental conditions, and have been shown to exhibit spatial clustering in contexts of environmental change [3,4]. This layer is explicitly bidirectional: alongside negative indicators (eco-anxiety, solastalgia, perceived threat), it incorporates positive affective and behavioral indicators, including restoration experience, place attachment, and soliphilia, a sense of care and responsibility toward one’s environment [13,32,33,34,35], understood as socially distributed and reinforced through collective practice rather than as an individual trait alone [36], consistent with evidence that direct, emotionally salient contact with nature is a primary driver of place attachment and connectedness [34,37]. This bidirectional structure allows EAHM to characterize eco-affective resource alongside eco-affective burden within the same spatial unit, rather than treating environmental well-being as merely the statistical absence of distress.

Integration across these layers is achieved through a structured spatial analytical pipeline. Environmental exposure variables, landscape configuration metrics, mobility-derived interaction profiles, and affective indicators are harmonized within a shared spatial grid. A formal specification of the resulting composite metric, the Eco-Affective Load Index (EALI), together with its constituent domain sub-indices, is provided in Section 4.1.

**Figure 2:**
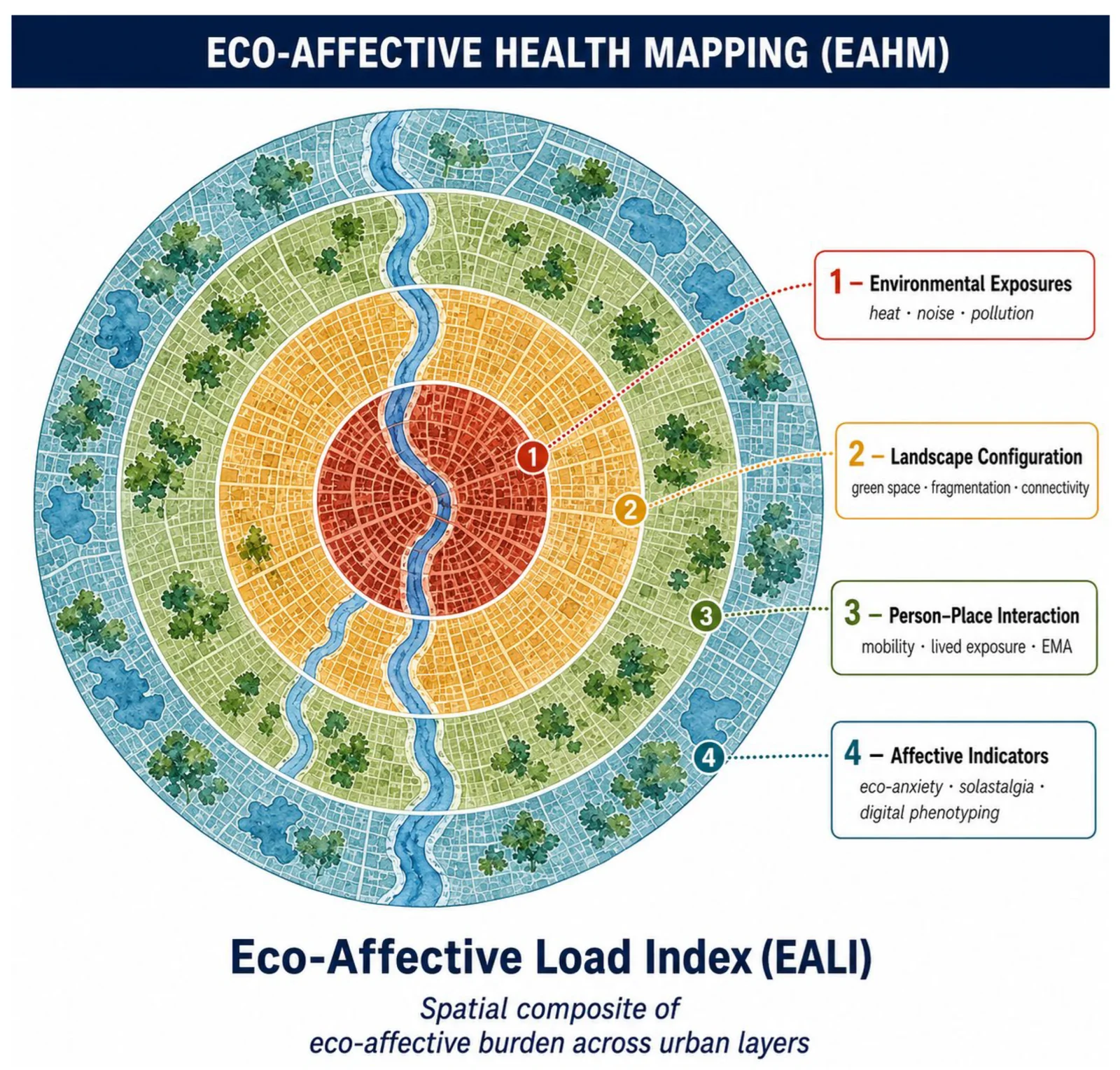
Conceptual structure of the Eco-Affective Health Mapping (EAHM) framework represented as a concentric spatial map. The map is organized into four rings, each corresponding to one analytical layer: (1) Environmental Exposures (innermost ring, terracotta), encompassing heat load, noise intensity, air pollution, and flooding risk; (2) Landscape Configuration (amber ring), reflecting green space distribution, habitat fragmentation, built-form density, and ecological connectivity; (3) Person–Place Interaction (moss green ring), capturing mobility patterns, transit and pedestrian infrastructure, lived exposure sequences, and ecological momentary assessment data; and (4) Affective Indicators (outermost ring, petrol blue), including eco-anxiety, solastalgia, soliphilia, place attachment, and digital phenotyping signals. All four layers converge into the Eco-Affective Load Index (EALI), reported as a four-domain profile alongside its composite score, as specified in Section 4.1. Figure created by the author with the assistance of a generative AI image-generation tool (see Acknowledgments); conceptual structure and content specification are the author’s own.

By treating climate-related emotional responses as spatially structured public health signals, the EAHM framework enables the integration of eco-affective dimensions into urban health monitoring, surveillance, and planning systems. Figure 2 illustrates the integration of the four EAHM layers within a unified spatial framework, enabling the identification of environmental-response gradients and eco-affective vulnerability hotspots across urban systems.

### 4.1. Operationalizing the Eco-Affective Load Index (EALI)

To move from conceptual architecture to a measurable output, I provide a formal, preliminary specification of EALI. Because the four EAHM layers are conceptually and empirically heterogeneous, biophysical exposure metrics, landscape structure metrics, mobility and interaction metrics, and self-reported or physiological affective indicators do not share a common metric and are not guaranteed to co-vary in a single direction, I propose, consistent with the domain-composite scoring logic developed for the companion EAHAP protocol [16], that EALI be constructed and reported as a multi-domain profile rather than collapsed prematurely into a single undifferentiated scalar.

For a given spatial unit i (for example, a census tract) and observation window t, each layer generates a domain sub-index computed as the mean of z-standardized indicators within that layer:

EEIi,t (Environmental Exposure Index) = mean of z-scored heat load, air pollution, noise, and vegetation-deficit indicators.

LCIi,t (Landscape Configuration Index) = mean of z-scored fragmentation, connectivity, density, and built-form indicators (reverse-scored so that higher values indicate less restorative structure).

PPIIi,t (Person-Place Interaction Index) = mean of z-scored exposure-sequence and mobility/accessibility indicators, capturing both stressor encounter and avoidance opportunity.

AAIi,t (Affective and Behavioral Index) = mean of z-scored negative eco-affective indicators (eco-anxiety, solastalgia, threat perception) minus mean of z-scored positive indicators (restoration experience, place attachment, soliphilia).

The composite EALI is then defined as a weighted sum: EALIi,t = w1·EEIi,t + w2·LCIi,t + w3·PPIIi,t + w4·AAIi,t, where the weights sum to 1.

Equal weighting (w1 = w2 = w3 = w4 = 0.25) is proposed as a transparent, assumption-light default, analogous to the equal-weighting convention adopted for domain composites in the EAHAP protocol [16]; studies with adequate data are encouraged to report empirically or expert-weighted composites as sensitivity analyses rather than as replacements for the default. Critically, I recommend that the four domain sub-indices always be reported and interpreted alongside the composite, not in place of it, because a single scalar cannot distinguish, for example, a unit with high stressor exposure but strong protective landscape structure from a unit with low exposure but poor person-place accessibility. The composite is intended as a screening summary; the sub-index profile is the diagnostic layer beneath it, directly addressing the concern that a single Eco-Affective Load Index cannot, on its own, capture the heterogeneity of the four underlying domains.

### 4.2. Illustrative Application (Hypothetical, Non-Empirical)

To illustrate, rather than validate, how EALI could function in practice, I describe a purely hypothetical worked example set in a mid-sized Brazilian city. Consider two adjoining neighborhoods: Bairro A, a densely built, low-vegetation district bordering an arterial road, and Bairro B, a lower-density district with substantial tree canopy and a nearby urban park. Under illustrative parameter values only, Bairro A might show an elevated Environmental Exposure Index (high heat and noise loading), a Landscape Configuration Index reflecting fragmented, low-connectivity green structure, a Person-Place Interaction Index reflecting limited pedestrian and transit alternatives to arterial-road exposure, and an Affective and Behavioral Index weighted toward eco-anxiety and low restoration experience, together summing to a high composite EALI. Bairro B, under the same illustrative logic, would be expected to show a lower composite EALI, driven primarily by its Landscape Configuration and Affective and Behavioral sub-indices. This example is presented solely to demonstrate how the four sub-indices and the composite would be computed and interpreted jointly; it does not represent observed data, and the specific values, directions, and comparative conclusions it implies are hypothetical illustrations that require empirical confirmation under the measurement strategy outlined in Section 5, not empirical findings in themselves.

## 5. Methodological Considerations for Measurement

Because EAHM is proposed here as a conceptual and spatial-analytical architecture rather than as a validated measurement instrument, its empirical application requires a defined measurement strategy for each layer. Specifying this strategy, even in outline, is necessary to make the framework testable rather than merely descriptive.

For the affective and behavioral layer specifically, I recommend the use of a standardized, modular instrument battery rather than ad hoc or single-study measures, in order to support cross-study and cross-city comparability. The companion Eco-Affective Health Assessment Protocol (EAHAP) [16] provides such a battery, organizing predominantly validated self-report instruments for solastalgia, eco-anxiety, ecological grief, place attachment, and related constructs into domains that map directly onto the affective and behavioral layer of EAHM, together with a defined administration order, a cultural-adaptation procedure, and explicit missing-data handling rules.

Spatial units for EAHM implementation should be defined at a resolution that balances geographic specificity with participant privacy, for example the census tract (setor censitário in the Brazilian context) or an equivalent administrative unit, consistent with the aggregation approach used for spatial linkage in the EAHAP protocol [16]. Temporal frequency of assessment depends on study objective: cross-sectional implementations can characterize a single EALI snapshot per spatial unit, while studies aiming to test the hypothesized early-warning function of eco-affective indicators require repeated or ecological-momentary assessment (for example, 30-, 60-, or 90-day exposure windows), supporting dose-response and change-point analyses analogous to those proposed for the broader Eco-Affective Health framework [13]. Analytical procedures should include spatial autocorrelation and hot-spot detection methods (for example, Local Moran’s I or Getis-Ord Gi*) applied to each domain sub-index and to the composite EALI, together with sensitivity analyses examining whether spatial clustering patterns are robust to alternative sub-index weighting schemes, an approach conceptually analogous to the use of early-warning signals for critical transitions in complex socio-ecological systems more broadly [38].

I emphasize that none of these measurement procedures have yet been applied empirically within EAHM at the time of writing. Their specification here is intended to make the framework operationally testable, not to report results of testing that has already occurred.

## 6. Applications for Global Public Health

The EAHM framework provides a scalable approach for integrating environmental monitoring with climate-related emotional response indicators within urban public health systems, particularly in rapidly urbanizing regions. By capturing spatial patterns of eco-affective burden, EAHM enables the identification of populations and neighborhoods at elevated risk of climate-related mental health outcomes, supporting proactive surveillance and prevention-oriented interventions rather than reactive, outcome-based responses [7,8].

From a public health policy perspective, EAHM offers a structured framework for translating environmental data into actionable insights for mental health decision-making. By spatially integrating environmental exposures with eco-affective indicators, the framework enables policymakers to identify communities where cumulative environmental stress may generate elevated eco-anxiety, solastalgia, or ecological grief before more visible mental health system disruptions emerge. This allows interventions, including green infrastructure expansion, heat mitigation strategies, noise regulation, and the establishment of community-based mental health resilience programs, to be prioritized not only based on physical exposure thresholds, but also on their projected influence on population eco-affective health.

Urban planning and environmental policy represent primary domains of application. Cities undergoing rapid expansion experience simultaneous increases in heat exposure, vegetation fragmentation, and noise intensity, factors associated with amplified climate-related emotional responses and reduced psychological resilience [9,10]. By mapping the spatial distribution of these exposures alongside eco-affective indicators, EAHM supports the identification of priority zones for interventions such as tree canopy expansion, restoration of ecological corridors, and redesign of public spaces to enhance population-level mental health and restoration capacity. Because EAHM captures relative eco-affective load, it provides actionable insights even in contexts with limited access to formal mental health infrastructure.

The Brazilian context illustrates the framework’s applicability in a high-diversity, rapidly urbanizing setting. Brazil combines high climatic heterogeneity with pronounced socio-spatial inequalities, resulting in uneven distributions of environmental exposures and mental health resources. National datasets such as DATASUS, combined with remote sensing data from INPE and land-use monitoring systems, allow the integration of environmental variables with spatially resolved indicators of eco-affective health. Previous research has demonstrated that responses to environmental degradation, including solastalgia, exhibit geographic clustering in regions undergoing rapid ecological change [3], and that urban heat and green space deprivation are associated with differential mental health outcomes across municipalities [10]. Integrating these dynamics within the EAHM framework enables the identification of socio-ecological vulnerability hotspots where environmental interventions may produce disproportionate benefits for population mental health. As with the rest of the framework, this application remains conceptual until tested empirically using the census-tract-level linkage procedure outlined in Section 5.

Importantly, EAHM is designed to be transferable across diverse urban and socioeconomic contexts, including data-constrained environments. While high-resolution datasets enhance analytical precision, the framework does not depend on comprehensive clinical infrastructures. Proxy indicators derived from remote sensing, open environmental databases, and low-burden affective measures can be integrated to approximate eco-affective load at the population level. This flexibility enables incremental implementation in rapidly urbanizing regions and Global South contexts, while preserving the framework’s core spatial and public health logic.

**Figure 3:**
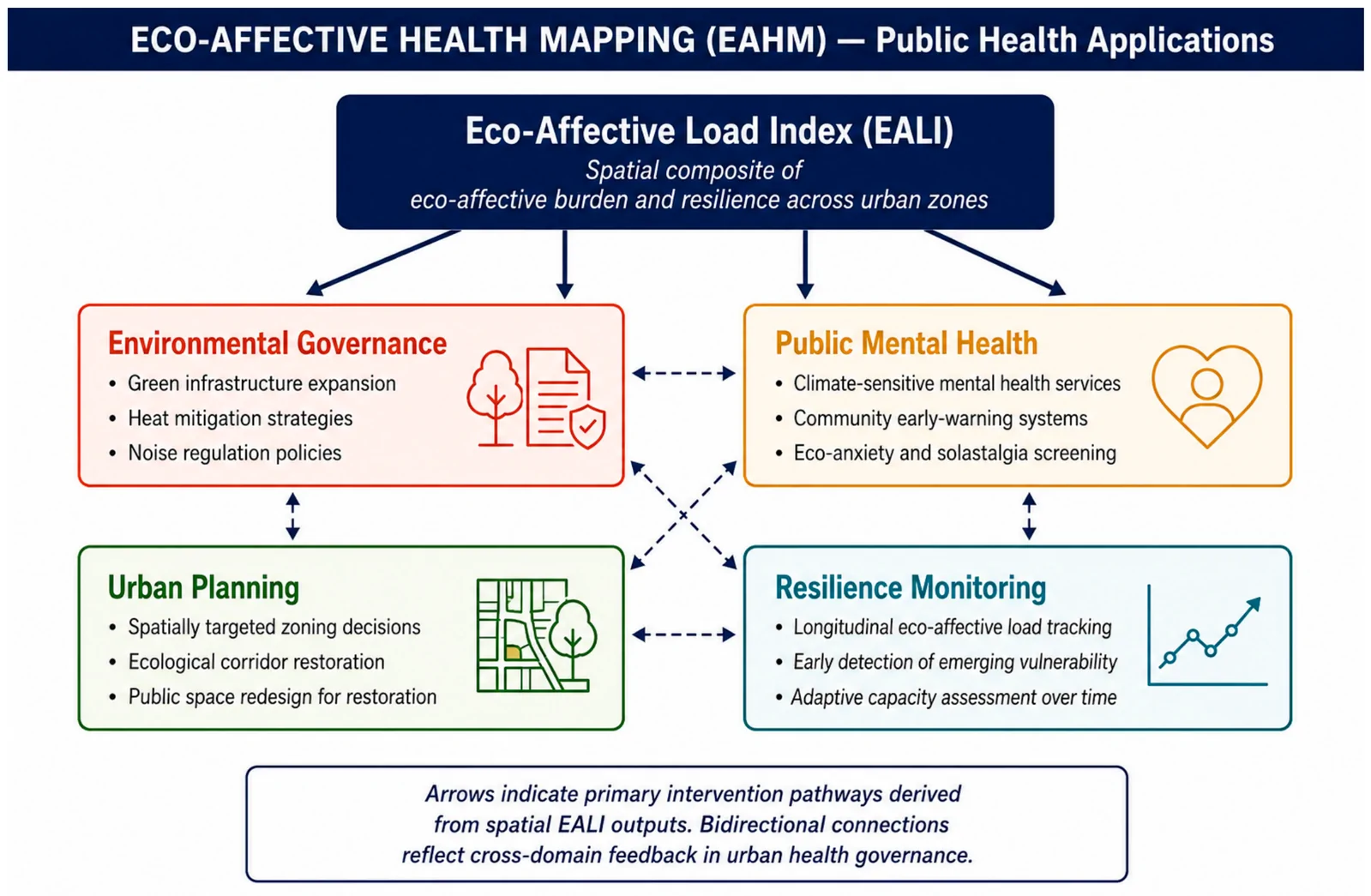
Translation of Eco-Affective Health Mapping (EAHM) outputs into public health applications. The Eco-Affective Load Index (EALI) serves as the central spatial output, informing four interconnected intervention domains: Environmental Governance (green infrastructure expansion, heat mitigation, noise regulation); Public Mental Health (climate-sensitive mental health services, community early-warning systems, eco-anxiety and solastalgia screening); Urban Planning (spatially targeted zoning, ecological corridor restoration, public space redesign); and Resilience Monitoring (longitudinal eco-affective load tracking, early detection of emerging vulnerability, adaptive capacity assessment). Bidirectional dashed arrows indicate cross-domain feedback processes in urban health governance. Figure created by the author with the assistance of a generative AI image-generation tool (see Acknowledgments); conceptual structure and content specification are the author’s own.

The framework also supports monitoring across temporal scales. As climatic conditions, vegetation cover, and urban form evolve, EAHM can track shifting gradients of eco-affective burden and population resilience, providing early signals of emerging mental health vulnerability. The use of digital phenotyping and ecological momentary assessment further enables continuous and fine-grained monitoring of climate-related emotional responses without reliance on large-scale institutional health infrastructures [11,12]. Figure 3 illustrates how EAHM outputs translate into four interconnected public health intervention domains.

## 7. Challenges and Future Directions

The implementation of EAHM requires addressing several methodological and infrastructural challenges that reflect the complexity of integrating eco-affective health indicators into public health monitoring systems. A key challenge lies in the heterogeneity of environmental data infrastructures. Remote sensing products, municipal land-use datasets, and satellite-derived exposure metrics vary in spatial and temporal resolution and in processing pipelines [10]. Establishing multi-scale harmonization standards will be essential to ensure comparability of eco-affective metrics across urban systems and to support valid cross-context inference.

A second challenge concerns the valid and equitable measurement of climate-related emotional responses across diverse populations. Instruments for assessing eco-anxiety, solastalgia, and ecological grief have advanced considerably in recent years, but their psychometric properties have not been validated uniformly across cultural, linguistic, and socioeconomic contexts [2,4]. Two of the instruments most relevant to solastalgia and ecological grief measurement remain explicitly flagged as candidate, rather than fully validated, adaptations pending independent psychometric confirmation [16]. Developing culturally adapted and low-burden measurement tools is a priority for ensuring that EAHM captures eco-affective health equitably, particularly in communities most affected by environmental change but least represented in existing research.

A third challenge involves governance and ethics. The integration of behavioral and affective indicators into spatial public health monitoring requires frameworks that ensure privacy, transparency, and data sovereignty, particularly in contexts historically affected by unequal data ownership and surveillance [8,39], and must additionally attend to the uneven distribution of environmental and eco-affective burden across populations, an equity concern raised more broadly in the environmental justice and climate-mental-health literatures [40]. Addressing these concerns is essential for the legitimate and equitable implementation of EAHM in public health practice.

These challenges, however, reflect a field transitioning toward operational maturity rather than conceptual limitation. Empirical evidence consistently demonstrates that environmental structure is associated with patterned variations in climate-related emotional responses across climatic and socio-ecological contexts [3,7,8]. The spatial clustering of eco-affective responses to environmental degradation indicates that these dynamics are closely tied to transformations within urban environments and can be meaningfully captured through spatial public health methods.

Looking forward, three strategic priorities emerge for advancing eco-affective health within public health science: (i) integration into urban health monitoring systems, incorporating eco-affective indicators alongside environmental and social determinants of health; (ii) translation into planning and policy, using eco-affective gradients to guide urban design, zoning, and climate adaptation strategies; and (iii) application in mental health resilience planning, identifying populations at elevated risk of eco-affective burden before more severe mental health disruptions occur. Above all of these, I regard prospective, multi-site empirical validation of the EAHM layer structure and the EALI specification proposed in Section 4.1, using the measurement strategy outlined in Section 5, as the most urgent next step, without which the priorities above cannot be responsibly pursued.

As urbanization, climate variability, and socio-environmental inequalities intensify, the capacity to spatially monitor climate-related emotional responses will become increasingly central to public mental health science. Eco-affective health should therefore be understood not as a peripheral extension of existing frameworks, but as a necessary expansion that integrates human experiential and emotional dimensions into the analysis and governance of urban environments under conditions of climate change.

## 8. Limitations

Before concluding, I state plainly the scope and limits of this contribution. EAHM is presented here as a theoretical and methodological framework: no environmental, mobility, or affective data were collected, analyzed, or reported in this article, and none of the relationships it proposes, including the four-layer structure, the EALI specification in Section 4.1, and the illustrative example in Section 4.2, should be read as empirically established. The framework’s constituent hypotheses, that eco-affective indicators cluster spatially in relation to environmental exposure and landscape structure, and that the four proposed sub-indices behave as distinct and jointly informative signals rather than as redundant restatements of a single underlying factor, require prospective empirical testing using the measurement approach outlined in Section 5 before EAHM can be considered validated for public health application. A further limitation concerns instrument availability: as noted in Section 7, some of the affective indicators specified for Layer 4 rely on adapted measures that are themselves pending independent psychometric validation [16], so early empirical applications of EAHM should treat their affective sub-index with corresponding caution. I regard prospective validation as the necessary next step for the research program this article proposes, not as a caveat to be resolved elsewhere by others.

## 9. Conclusions

Urban environmental stress is not only ecological and material but is also expressed through climate-related emotional responses that accumulate differentially across populations and places. Evidence increasingly demonstrates that eco-anxiety, solastalgia, ecological grief, and related affective states are spatially structured, shaped by landscape configuration, environmental exposures, and socio-infrastructural conditions [3,4,8]. The Eco-Affective Health framework builds on this understanding by integrating environmental metrics and eco-affective indicators into a unified spatial logic, proposed here, pending empirical validation, to enable the identification of gradients of mental health vulnerability and resilience across urban populations. By positioning climate-related emotional responses as candidate measurable public health signals, the framework advances the integration of ecological, affective, and spatial dimensions within a single analytical approach intended for future application to urban planning, climate adaptation, and population mental health surveillance.

Rather than replacing planetary or environmental health perspectives, Eco-Affective Health extends them by incorporating human experiential and emotional signals as candidate real-time indicators of how urban systems affect population mental health under conditions of environmental change. As cities continue to expand under conditions of climate instability, the ability to map and interpret these dynamics is expected to become essential for guiding prevention-oriented, equitable, and sustainable interventions that protect both human well-being and environmental integrity, provided the framework’s core claims are first confirmed through the empirical program outlined in Section 5 and Section 8.

## Data Availability

No new data were created or analyzed in this study. Data sharing is not applicable to this article.
